# The Treatment of the Uterine Displacements

**Published:** 1892-05

**Authors:** Frank W. Patch

**Affiliations:** South Framingham, Mass.


### THE TREATMENT OF THE UTERINE DISPLACEMENTS.

FRANK W. PATCH, M. D., SOUTH FRAMINGHAM, MASS.

The causes leading to displacements of the female sexual organs are briefly cited by Dr. Southwick in his recent work on Gynæcology, as follows:

1. Lack of sufficient support to the uterus.
   *a.* Laceration of the perineum.
   *b.* Relaxation of the vagina.
   *c.* Weakness of the uterine ligaments.

2. Morbid conditions of the uterus.
   *a.* Tumors.
   *b.* Chronic congestion.
   *c.* Hyperplasia.
   *d.* Lack of tone or relaxed condition of the uterine tissue.

3. Force acting on the uterus from above.
   *a.* Tight or heavy clothing supported from the abdomen.
   *b.* Tumors or a distended bladder crowding the uterus out of place.
   *c.* Bad positions while sitting in easy chairs.
   *d.* Straining at stool.
   *e.* Falls or jumping.
   *f.* Undue exercise.

4. Traction on the uterus.
    *a*. Dragging of a prolapsed vagina.
    *b*. Abnormally short vagina.
    *c*. Cicatrices or contracting masses of lymph.

Aside from cases of congenital malformation and those influenced by improper clothing or the displacement from crowding by pathological growths, we would still more briefly state that the causes leading to displacements of the female genital organs seem to lie, practically, wholly in a loss of the proper muscular tonicity and strength, either of the organs themselves or of the surrounding and sustaining structures, or both, resulting from countless conditions of ill health, as many and different as we have individuals to deal with. These conditions are made manifest to the physician in the same manner as is sickness in any other part of the human system, viz., by a train of symptoms more or less severe according to the circumstances of the case, and in that train of symptoms, we, as homœopathists, should find the true pathology of the condition, and be led to undertake a cure in the same manner as though we were dealing with an affection of the muscles of the arm or back or other part of the body. Just why the appearance of a patient exhibiting a peculiar group of symptoms supposed to indicate a displacement should cause us to forsake our universal means of cure in a vain search for some shape of pessary or combination of cerate with which to pursue treatment is one of the mysticisms of medicine. It savors too much of Kochism or Brown-Sequardism to be a pleasant theme for contemplation by Homœopathy. Indeed! do we not resemble, in this, that eminent one who throughout his long life sought up and down the world for the "Great Stone Face" of Nature, only to find at the end that the face was more nearly depicted in his own thought than elsewhere.

In an able paper by Dr. J. T. Kent, of Philadelphia, read before the I. H. A. last year, occur the following instructions in regard to the treatment of these cases: "Whenever a patient presents herself to the Hahnemannian physician for relief from the complexity of symptoms belonging to displacements, not only the symptoms of the displacement, but all the symptoms of the case, from childhood to the present time, must be as accurately written down as it is possible to obtain them, after the method directed by the *Organon*." "An examination, such as is generally given, is of the smallest importance and reveals none of the peculiar characteristics upon which the physician must rely for this symptom image." "The mechanical support (if one has been worn), must be removed at once, else the symptoms of most value do not appear, the patient then should be placed upon Sac-lac. for at least a week before a full symptom image will be found."

Thus we see that here, as elsewhere, we are to place our reliance on the group of symptoms elicited as a guide to the treatment of these cases, on the individualization of the case and consequent search through the materia medica for the most appropriate remedy, leaving to those who have not better means the use of that hobgoblin of practice—the pessary. And by so doing we shall not only gain more success in treatment but also the wholesome gratitude of our patients, the delicate women, who find themselves obliged to submit to the grossly disagreeable measures involved, in the use of such mechanical methods of treatment. Indeed, they should learn that Homœopathy has something better to offer them, that they need not undergo a treatment, the very knowledge of which deters many a woman from seeking our aid, and thereby condemns her to life-long torture. And were it universally so understood, how long would it be before our offices would claim a majority of this class of cases?

Are we not pursuing a short-sighted policy in more ways than one by wandering from our long-tried and legitimate means of treatment?

In glancing through our materia medica one is surprised to learn that we have there some sixty remedies at least, the pathogeneses of which contain symptoms related in a greater or less degree to the conditions of which we have been speaking. In the hope that a compact arrangement of these symptoms may be of use to some in the hurry of daily practice, I have tabulated this list alphabetically, knowing, of course, that it can serve simply as a possible index which may aid in fixing on the remedy containing the totality of symptoms in a given case. As the entire list would prove but tedious reading at this time, it may be well to point out only the prominent characteristics of some of the more important remedies. This secondary list, which, by the way, is of primary importance, might include Aurum, Belladonna, Helonias, Lilium-tig., Murex, Nux-vom., Podophyllum, Pulsatilla, Sepia, Ustilago, and these remedies will, perhaps, cover the majority of cases which come to our notice.

In Aurum we find the uterus prolapsed and indurated, bruised pain, with shooting and drawing ; heaviness in abdomen ; after lifting a heavy load (Pod., Arn., Rhus-tox, Nux-v.) , worse at time of menses. Amenorrhœa with prolapsus uteri and melancholy.

Belladonna, a violent pressing and urging toward the genitals as if everything would fall out there ; in Sepia the bearing-down pains begin in the back and the patient is impelled to cross the limbs to prevent protusion of the parts (in Nat-mur. she must sit down to prevent protrusion of the parts). Perhaps Belladonna and Sepia will be indicated at different stages of the same case, if so, the former will usually be found more appropriate in the earlier or acute stage, the latter after more advancement or when the Bell. seems to cease its beneficial action. The Belladonna patient also is distinguished by many other symptoms peculiar to herself alone ; she is worse while sitting bent and in walking ; better on standing and sitting erect (Rheum. is worse while standing). Then, too, Belladonna has clutching and clawing pains or stitches in the uterine region ; the pains of Sepia are stitching, shooting, or lancinating, mostly in the neck of the uterus, and they go up to the umbilicus or pit of stomach. The Belladonna patient cannot bear the least jar (Lil-t.) ; great heat and dryness of the vagina. Pressure downward, as if all the contents of the abdomen would issue through the vulva ; worse mornings. The first part of the above symptom is often met with in these cases and may also be found under several other remedies, as Actea, Agaricus, Antimonium-crudum, Apis-mel., Asafœtida, Cantharis, Conium, Ferrum, Graphites, Kreosote, Lilium-tigrinum, Murex, Natrum-carb., Natrum-muriaticum, Nitric acid, Nux-vomica, Rheum, Rhus-tox., Sepia, Silicea, Stannum, Sulphur, Ustilago.

Yet in only two other of these (Nat-mur., Nux-vom.) do we find the condition aggravated in the morning, and even here is the beauty of our system of individualization apparent when we notice that the Natrum-muriaticum patient must sit down to prevent prolapsus, while the Belladonna patient is worse when sitting, unless bolt upright, and is better when standing. The morning aggravation in Nux-vomica is more of a general condition.

Under Asafœtida we find this bearing-down sensation with the additional peculiarity of aggravation when riding in a carriage. These conditions of aggravation and amelioration, as is well known, are of the highest importance, and often form the pivot on which will turn our choice in the selection of the proper remedy. The sensation that the contents of the abdomen are coming through the vulva is peculiar, though not in general uncommon, and, indeed, the cervix often does protrude or nearly so in very relaxed conditions.

The remedies beside Belladonna having this symptom in some form are Antimonium-crudum, Lilium-tigrinum, Natrum-carb., Natrum-mur., Nitric acid, Podophyllum, Sepia, and Ustilago.

In Lilium-tig. the sensation is relieved by the pressure of the hand against the vulva; in Natrum-mur. by sitting down; in Podophyllum it occurs during stool, and in Sepia is accompanied with oppressed breathing.

Helonias may be brought to our minds by the mental condition of the patient, which is often prominent; she is overcome by a profound melancholy and deep, undefined depression; this you will notice might also suggest Aurum were it not for the fact that the Helonias patient is better when her mind is engaged, while the Aurum patient is fatigued by mental occupation, and may also exhibit the further characteristic of suicidal desire.

With Helonias there is also a distinct sensation of soreness and weight in the womb, expressed as a consciousness that she has a womb. Dragging weakness in the sacral region with prolapsus; also at climaxis with maked debility and profound mental gloom. Prolapsus uteri and ulceration of the cervix, discharges constant, dark, badly smelling (Arg-nit., Plat.); flooding on lifting a weight, and on least exertion; face sallow, having an expression of suffering; great vaginal irritation; pain in small of back; prolapsus uteri with leucorrhœa; the os protrudes externally; uterus low down, fundus tilted forward; the finger passes with difficulty between the os and the rectum; great uterine hemorrhage; pain in back through to uterus.

The above picture will be recognized as applying to a class of cases which may well try the patience of physician and sufferer. The woman who after years of treatment by medicines, local applications, the various styles of pessaries, and finally surgery has at last become reduced to a state of chronic invalidism, and leads a miserable existence without hope of comfort in this world. Who wonders that she is skeptical of the power of Homœopathy to bring light to her dark horizon. She is literally worn out, either by hard work or by enervating luxury and physicians. The most characteristic symptom is that her condition is ameliorated when her mind is occupied, hence during the physician's visit. Dr. Samuel A. Jones says of Helonias, that the "headache disappears when the attention is engaged. The pains vanish when one is busied. The sense of profound debility is lost when exercising." It acts by opposites.

In Lilium-tigrinum we have bearing-down pains in the uterus, with pains in the left ovary and left mamma. Severe neuralgic pains in uterus so that she cannot bear touch, not even the weight of the bed-clothes, or slightest jar; anteversion; this much resembles Belladonna, except that these symptoms of Lilium are apt to be worse in the afternoon, those of Belladonna in the morning. Aching over the pubes, with pain in knees. Bearing down in uterine region as if everything would be pressed out; relieved by pressure of hand against vulva. Bloated feeling in the region of uterus; pelvic organs feel swollen; the aching seems to be around, and not in the uterus; intermittent sharp pains across the lower bowels. Intermittent labor-like pains (Acon., Asafœt., Kali-carb., Nux-mos.), in lower part of back, with a thin, acrid, excoriating leucorrhœa, leaving a brown stain; worse afternoons till twelve P. M. Pressure in anterior wall of rectum, with bearing down, low spirited, weeping, apprehensive, irritable, opposite and contradictory mental states (Helonias). Urgent desire for stool; anorexia; faint in a close room, and when standing; frequent, scant, and burning urine, pain in sacrum, bloated feeling in abdomen, limbs cold and clammy.

*Murex.*—Dragging down, desire for external pressure over the parts, with awful sense of hunger in the stomach, even after eating; emptiness, goneness, and sinking in the stomach, lingering constipation, with a sexual instinct that drives her frantic.

*Nux-vomica.*—Pressure toward the genitals in the morning; prolapsus uteri, from overlifting or straining, bearing down toward the sacrum, with ineffectual urging to stool; burning, heaviness, sticking in the uterus.

*Podophyllum.*—Prolapsus uteri after overlifting or straining, after parturition, with pain in sacrum, rumbling in ascending colon; prolapsus ani or constipation. Sensation as if genitals would come out during stool. (Calcarea-phos. has an aggravation during the passage of the stool and urine.) Suppressed menses in young females, with bearing down in hypogastric and sacral regions, with pain from motion, better lying down (Nat-mur., better lying on back).

*Pulsatilla.*—Prolapsus uteri, with pressure in the abdomen and small of back, as from a stone; limbs tend to go to sleep, ineffectual urging to stool (Nux-v.)

*Sepia.*—Pain in the uterus, bearing down, comes from back to abdomen, causing oppression of breathing; she crosses her limbs to prevent protrusion of the parts; stitching, shooting, lancinating pains, mostly in the neck of uterus, go up to umbilicus and pit of stomach. Prolapsus of the uterus, of the vagina, with constipation; induration of the neck of the uterus.

*Ustilago.*—Constant aching, referred to the mouth of the womb; displaced uterus, with menorrhagia; cervix tumefied, bleeds when touched; for days oozing of dark blood, with small coagula; uterus enlarged, cervix tumefied or dilated. Bearing down, as though everything would come through.

Aconite.—Prolapsus uteri, occurring suddenly, with inflammation, bitter vomit, cold sweat, or dry, hot skin. Labor-like pressing in the womb; has to bend double, but relieved in no position.

Actea-rac.—Bearing down in uterine region and small of back, limbs feel heavy, torpid. Leucorrhœa, with sensation of weight in the uterus. Severe pain in lower part of abdomen, worse by motion.

Agaricus.—Prolapsus uteri after cessation of menses. Bearing-down pain almost intolerable, menses too profuse, with tearing, pressive pains in back and abdomen.

Aloe-soc.—Fullness, heaviness in uterine region, with labor-like pains in loins and groins.

Ammo-mur.—Menses too early, with pain in abdomen and small of back, continuing at night; flow more profuse at night; prolapsus uteri.

Ant-crud.—Pressure in the womb as if something would come out.

Apis-mel.—Feeling of weight, heaviness in the ovarian region, great tenderness over the uterine region, with bearing-down pain, leucorrhœa, and painful urination. Heat and fullness of uterine region. Burning or stinging pain in the region of uterus or ovaries.

Arg-met.—Prolapsus uteri; pain in left ovary; pain in small of back, extending to the front and downwards.

Arg-nit.—Prolapsus with ulceration of os or cervix uteri. Leucorrhœa copious, yellow, corroding.

Arnica.—Prolapsus caused by concussion.

Asafœtida.—Labor-like pains in uterine region, with cutting and bearing down. Bearing down in genitals; worse when riding in a carriage.

*Aurum.*—Uterus prolapsed and indurated, bruised pain, with shooting and drawing, heaviness in abdomen; after lifting a heavy load; worse at time of menses. Amenorrhœa with prolapsus uteri and melancholy.

*Belladonna.*—Violent pressing and urging toward the genitals as if everything would fall out there; worse sitting bent, and in walking, better standing and sitting erect. Clutching, clawing pains or transient stitches in the uterine region, parts sensitive, cannot bear least jar. Pressure downwards as if all the contents of the abdomen would issue through the vulva; worse mornings; great heat and dryness of vagina.

Benz-acid.—Prolapsus uteri with fetid urine.

Bovista.—Painful bearing down in vulva and weight in small of back, after midnight.

Cactus.—Painful constriction around the pelvis, extending gradually toward the stomach, causing a sensation as of a great blow in the region of the kidneys, making her cry out. Pulsating pain in ovarian regions; pains extend down thighs, return periodically at the same time each day.

Calcarea-carb.—Inflammation and swelling of the genitals. Uterus easily displaced by overexertion.

Calcarea-phos.—Throbbing, stinging, tickling, sore aching or pressing in the gentials, drawing upward in the symphysis, downward in the thighs. Weakness and distress in the region of the uterus, worse during passage of stool and urine.

Cantharis.—Ovarian region; stitches, arresting the breathing; violent pinching pains, with bearing down toward the genitals; great burning pain.

Carbo-an.—Tearing transversely across the pubes and then through pudenda, as far as the anus. Induration of neck of uterus; burning.

Menorrhagia from chronic induration of uterus; also in delicate women, with glandular affections.

Caulophyl.—Sensation as if the uterus was congested, with fullness and tension in the hypogastric region. Menstrual colic; uterus retroverted.

Cinchona-off.—Congestion to the uterus, fullness, pressing and heaviness, worse when walking.

Conium.—Stinging in the neck of uterus. Induration and prolapsus at the same time. Burning, sore aching sensation in the region of the uterus. Pressure from above downwards and drawing in legs during menses.

Ferrum.—Sharp pains in abdomen, bearing down in uterus, with aching below it. Stitching, shooting pains in uterus. Pains near mouth of uterus when lying down.

Gels.—Uterus as if squeezed by a hand, anteflexion.

Graphites.—Tumor, size of an orange, right iliac fossa, also similar one in left; both hard, round, slightly movable; not painful to pressure, nor producing inconvenience from weight. Os uteri standing backwards, can only be reached with difficulty. Pain in the uterus when reaching high with the arms. Bearing-down pain in the uterus, to back, with weakness and sickness. Painful pressure toward the pudenda. During the menses, heat in the abdomen, urging, pressing, like labor pains; hoarseness, lassitude, and weakness.

*Helonias-dioica.*—Profound melancholy, deep, undefined depression, with a sensation of soreness and weight in the womb; "a consciousness of a womb." Dragging weakness in sacral region, with prolapsus; also at climaxis, with marked debility; profound mental gloom. Prolapsus uteri and ulceration of the cervix; discharge constant, dark, badly smelling; flooding on lifting a weight and on least exertion; face sallow, having an expression of suffering; great vaginal irritation; pain in small of back. Prolapsus uteri, leucorrhœa; the os protrudes externally. Uterus low down, fundus tilted forwards; the finger passes with difficulty between the os and the rectum. Great uterine hemorrhage; pain in back through to uterus.

Hyos.—Uterine cramps, with pulling in loins and small of back; irritable uterus.

Iodum.—Induration and swelling of the uterus and ovaries. Dropsical affections of the ovaries, with pressing down toward the genitals. Dull, pressing, wedge-like pains, from right ovary toward the uterus.

Ipecac.—Prolapsus and hemorrhage at the menstrual period.

Kali-bich.—Prolapsus uteri, seemingly from hot weather. Leucorrhœa yellow, ropy; pain and weakness across the small of the back, and dull, heavy pains in the hypogastrium.

Kali-carb.—Stitching pains in and about the uterus, labor-like colic, leucorrhœa; pain like a weight in small of back.

Kreos.—Bearing down and weight in pelvis, painful urging toward the genitals; fundus of uterus swollen and sensitive to pressure.

Lachesis.—The uterine region feels swollen, will bear no contact, not even of the clothing; bearing-down pains. Pains like a knife thrust into abdomen.

*Lil-tig.*—Bearing down in uterus, pains in left ovary and left mamma. Severe neuralgic pains in uterus, could not bear touch, not even weight of bed-clothes or slightest jar; anteversion. Aching over the pubes, with pains in knees. Bearing down in uterine region, as if everything would be pressed out; relieved by pressure of hand against vulva. Bloated feeling in region of uterus; pelvic organs feel swollen; aching apparently around, not in, the uterus. Intermittent sharp pain across lower bowels. Burning from groin to groin, with morning stool. Intermittent labor-like pains in lower part of back, with a thin, acrid, excoriating leucorrhœa, leaving a brown stain; worse afternoon till twelve P. M. Pressure in anterior wall of rectum, with bearing down; low spirited, weeping, apprehensive, irritable; opposite and contradictory mental states; urgent desire for stool; anorexia; faint in close room and when standing; frequent, scant, burning urine; pain in sacrum; bloated feeling in abdomen, limbs cold, clammy.

Mag-mur.—Bearing down in the ovarian region.

Merc.—Deep, sore pain in the pelvis; dragging in the loins; abdomen weak, as if it had to be held up; prolapsus uteri and vagina; feels better after coitus.

*Murex.*—Dragging down, desire for external pressure over the parts, with awful sense of hunger in the stomach even after eating, which has an emptiness, a goneness, a sinking; lingering constipation with a sexual instinct that drives her frantic.

Nat-carb.—Pressure in hypogastrium, as if everything would come out; also with indurated and ill-shaped os.

Nat-mur.—Every morning pressing and pushing toward the genitals, must sit down to prevent prolapsus. Prolapsus uteri, with aching in lumbar region, better lying on back; also, with cutting in urethra after micturition.

Nit-ac.—Pressing down in hypogastrium and small of back, as though everything would protrude; pain down thighs, abdomen feels swollen.

Nux-mos.—Spasmodic labor-like pains, uterus displaced; mouth and throat dry, sleepy, faint; abdomen enormously distended after a meal, pressure in back outward. Sensation of a lump in left lower abdomen, anteversion. Prolapsus uteri et vaginæ.

*Nux-vom.*—Pressure toward the genitals in the morning; prolapsus uteri; from straining or lifting; bearing down toward the sacrum, with ineffectual urging to stool. Burning, heaviness, sticking in the uterus.

Opium.—Prolapsus uteri from fright.

Petroleum.—Prolapsus uteri in patients reduced by chronic diarrhœa, occurring during the day.

Platina.—Painful sensitiveness and continual pressure in region of mons veneris and genital organs; body, except the face, feels cold. Prolapsus uteri, induration of the uterus; ulceration, with existing ovarian irritation.

*Pod.*—Prolapsus uteri, after overlifting or straining; after parturition, with pain in sacrum; rumbling in ascending colon; prolapsus ani or constipation. Sensation as if genitals would come out during stool.

Suppressed menses in young females, with bearing down in hypogastric and sacral regions, with pain from motion; better lying down.

*Puls.*—Prolapsus uteri, with pressure in abdomen and small of back, as from a stone; limbs tend to go to sleep; ineffectual urging to stool.

Rheum.—Bearing down in uterine region while standing.

Rhus-tox.—Bearing down, when standing or walking, backache, better lying on something hard; prolapsus from overlifting or straining.

*Sepia.*—Pain in uterus, bearing down, comes from back to abdomen, causes oppression of breathing, crosses limbs to prevent protusion of parts. Stitches or shooting, lancinating pains, mostly in neck of uterus, go up to umbilicus and pit of stomach. Prolapsus, of the uterus, of the vagina, with constipation. Induration of neck of uterus.

Silicea.—Prolapsus uteri from myelitis. Pressing-down feeling in vagina; parts tender to touch.

Stannum.—Bearing down in uterine region; prolapsus uteri. Displacement of vagina; worse during stool; feels so weak she must drop down suddenly, but can get up quite readily.

Sulph.—Bearing down in pelvis, toward genitals. Labor-like pain over the symphysis. Weak feeling in genitals.

Sulph-acid.—Prolapsus of vagina; parts look greenish and smell badly.

*Ustilago.*—Constant aching, referred to mouth of womb. Displaced uterus, with menorrhagia; cervix tumefied, bleeds when touched. For days oozing of dark blood, with small coagula; uterus enlarged, cervix tumefied or dilated. Bearing down as though everything would come through.

Verat-alb. Strangulated prolapsed vagina with cold sweat, exhausting vomiting and diarrhœa. Dysmenorrhœa with prolapsus; vomiting, diarrhœa, exhaustion.

